# Human rotavirus strains circulating in Venezuela after vaccine introduction: predominance of G2P[4] and reemergence of G1P[8]

**DOI:** 10.1186/s12985-017-0721-9

**Published:** 2017-03-21

**Authors:** Esmeralda Vizzi, Oscar A. Piñeros, M. Daniela Oropeza, Laura Naranjo, José A. Suárez, Rixio Fernández, José L. Zambrano, Argelia Celis, Ferdinando Liprandi

**Affiliations:** 1 Laboratorio de Biología de Virus, Centro de Microbiología y Biología Celular, Instituto Venezolano de Investigaciones Científicas (IVIC), Apdo. 21827, Caracas, 1020-A Venezuela; 2 Policlínica Metropolitana, Caracas, Venezuela; 3 Universidad de Carabobo-Sede Aragua, Maracay, Edo. Aragua Venezuela

**Keywords:** Rotavirus, Diarrhea, Vaccination, Genotypes prevalence, Phylogenetic analysis, Genetic evolution, Venezuela

## Abstract

**Background:**

Rotavirus (RV) is the most common cause of severe childhood diarrhea worldwide. Despite Venezuela was among the first developing countries to introduce RV vaccines into their national immunization schedules, RV is still contributing to the burden of diarrhea. Concerns exist about the selective pressure that RV vaccines could exert on the predominant types and/or emergence of new strains.

**Results:**

To assess the impact of RV vaccines on the genotype distribution 1 year after the vaccination was implemented, a total of 912 fecal specimens, collected from children with acute gastroenteritis in Caracas from February 2007 to April 2008, were screened, of which 169 (18.5%) were confirmed to be RV positive by PAGE. Rotavirus-associated diarrhea occurred all year-round, although prevailed during the coolest and driest months among unvaccinated children under 24 months old. Of 165 RV strains genotyped for G (VP7) and P (VP4) by seminested multiplex RT-PCR, 77 (46.7%) were G2P[4] and 63 (38.2%) G1P[8]. G9P[8], G3P[8] and G2P[6] were found in a lower proportion (7.3%). Remarkable was also the detection of <5% of uncommon combinations (G8P[14], G8P[4], G1P[4] and G4P[4]) and 3.6% of mixed infections. A changing pattern of G/P-type distribution was observed during the season studied, with complete predominance of G2P[4] from February to June 2007 followed by its gradual decline and the reemergence of G1P[8], predominant since January 2008. Phylogenetic analysis of VP7 and VP4 genes revealed a high similarity among G2P[4] and global strains belonging to G2-II and P[4]-V lineages. The amino acid substitution 96D → N, related with reemergence of the G2 genotype elsewhere, was observed. The G1P[8] strains from Caracas were grouped into the lineages G1-I and P[8]-III, along with geographically remote G1P[8] rotaviruses, but they were rather distant from Rotarix^®^ vaccine and pre-vaccine strains. Unique amino acid substitutions observed on neutralization domains of the VP7 sequence from Venezuelan post-vaccine G1P[8] could have conditioned their re-emergence and a more efficient dissemination into susceptible population.

**Conclusions:**

The results suggest that natural fluctuations of genotypes in combination with forces driving the genetic evolution could determine the spread of novel strains, whose long-term effect on the efficacy of available vaccines should be determined.

## Background

Human rotavirus (RV) is the most important cause of severe, acute gastroenteritis in infants and young children in both developed and developing countries. Until 2008, rotavirus infections were estimated to cause approximately 453,000 deaths per year worldwide in children aged <5 years, most of them living in low-income countries [1]. Two live RV vaccines (oral and attenuated), the monovalent human RV vaccine Rotarix^®^ (GlaxoSmithKline Biologicals, Rixensart, Belgium), containing a single human G1P[8] RV strain, and the pentavalent bovine–human, reassortant vaccine RotaTeq^®^ (Merck & Co., Inc., Whitestation, NJ, United States), were licensed in 2006. Both have demonstrated very good safety and efficacy profiles in large clinical trials mainly in the United States, Europe and Latin America [2–4]. The inclusion of rotavirus vaccine in the immunization programs was recommended by World Health Organization since 2007 in regions where a significant public health impact was anticipated [5], and later in 2009, the introduction in developing countries was proposed worldwide. The implementation in many countries of the world has demonstrated to reduce the disease burden of rotavirus-specific diarrhea and death related in several regions [6–8].

Venezuela was among the first developing countries to introduce RV vaccines into the national immunization schedules in 2006. Both vaccines have been available: the Rotarix^®^ is the RV vaccine of choice in the public health care sector, and therefore the most used, while the RotaTeq^®^ is obtainable only in private facilities. Although some data have shown decline in hospitalizations and deaths related to severe diarrhea for any causes in children under 5-years of age after RV vaccine introduction [9], a report from the Center for Disease Control and Prevention (CDC) indicated no substantial changes in the percentage of RV diarrhea cases in 2010 (31%) compared with 2006 (32%) in Venezuela, where coverage has fluctuated around 49% in the last decade [10]. On the other hand, vaccine efficacy has been shown to be remarkably lower in developing countries [11]. Strain surveillance is needed to assess the impact of immunization on the RV diarrhea incidence and the variability of the circulating viruses.

In the last two decades, RV molecular genotyping has provided valuable information about the diversity of RV outer capside (VP7 or “G”, and VP4 or “P”) and some nonstructural proteins from strains circulating throughout the world. Currently, 27 G genotypes and 37 P genotypes have been described [12, 13]. Extensive molecular epidemiological studies globally have indicated that only a small number of genotypes have prevailed: G1P[8], G2P[4], G3P[8] and G4P[8] have been the most important in humans worldwide. Since 1995, G9P[8] increased dramatically, being now considered the fifth globally important RV genotype [14–16], and G12P[8] is currently also being increasingly detected around the world [17, 18]. Other G types of animal origin such as G5, G6 and G8 have acquired epidemiological relevance in some geographical areas of Africa, Asia and South America [19–21].

Temporal and geographical changes in genotype prevalence patterns have been seen with periodic emergence of novel strains, particularly in developing countries [14, 22] where the evolutionary dynamics of RV are complicated by a greater diversity. Surveillance of RV infection conducted in the past two decades in Venezuela has showed also a broad diversity and temporal variations for the G and P types circulating, with alternating predominance of G1P[8], G3P[8] or G4P[8] [23–26] and moderate rates of circulation of G2P[4] strains, in addition to the emergence of G9P[8] and appearance of G8P[14] [25, 26]. The effects of such variability on the efficacy of the vaccines have to be elucidated, and more whether vaccination may lead to the replacement of vaccine-type strains. Reports from Brazil, Australia, Portugal and USA have described changes in RV genotype prevalence following vaccine introduction [27–31], but whether the pattern and distribution of the most prevalent RV strains could be modified by the immunologic pressure exerted by the vaccines remains uncertain. The emergence of RV genotypes that are not efficiently controlled by the immune response induced by the vaccine is a possibility [32], but additional studies are needed.

The main aim of this study was to estimate the prevalence of RV gastroenteritis and distribution of circulating G (VP7) and P (VP4) genotypes from clinical isolates causing symptomatic infections in children with diarrhea, living in the metropolitan area of Caracas, one year after the RV vaccination started in Venezuela. In addition, phylogenetic analysis based on VP7 and VP4 gene of some isolates collected during 2007-2008 was performed in comparison with pre-vaccination RV strains collected in 2003 and global reference strains. In order to examine the potential modifications under vaccine selective pressure, VP7 and VP4 amino acid sequences were also deduced and analyzed to verify the occurrence of eventual substitutions.

## Methods

### Stool collection, RV testing and controls

Between February 2007 and April 2008, fecal samples were collected from 912 children younger than 10 years of age, who were attended for acute diarrhea, defined as three or more liquid stools over a 24-h period, in a private clinical setting of a medical center in Caracas, Venezuela. Out of 912, 69% proceeded from children over 16 months of age at the starting time of the study, therefore not eligible for rotavirus vaccination.

Stool specimens were immediately screened for the presence of rotavirus by use of an immunochromatographic rapid test, Rota-Strip (Coris BioConcept, Gembloux, Belgium), according to the manufacturer’s instructions. The samples were stored at -20°C until successively tested.

Cell culture-adapted RV strains grown in MA-104 cells were used as control strains for G- and P-genotyping assays. G/P type designations are as recommended by the Rotavirus Classification Working Group [12] and Reoviridae Study Group of the International Committee on Taxonomy of Viruses. The study was cleared by the IVIC ethic committee. A written informed consent was obtained from the parents or legal guardians at enrollment.

### RNA extraction, gel electrophoresis and silver staining

Rotavirus positive samples detected by immunochromatographic test were further analyzed by polyacrylamide gel electrophoresis (PAGE) in a 7% gel following viral RNA genome extraction by phenol and chloroform-isoamyl alcohol treatment, and ethanol precipitation. Double strand RNA (dsRNA) segments of RV were separated by PAGE at room temperature for 3 h at 90 V and stained with silver nitrate as described previously [33]. The electrophoretic migration patterns (electropherotypes) of the RNA segments were also analyzed for further genetic characterization of the strains.

### G/P genotyping of rotavirus

Rotavirus G (VP7) and P (VP4) genotypes were determined as described previously [26]. For this purpose, nucleic acids were extracted from 5% fecal suspensions clarified supernatant using the QIAamp^®^ Viral RNA Mini kit (QIAGEN^®^, Hilden, Germany) according to the manufacturer’s instructions. The extracted RNA was reverse transcribed and G and P genotyping was performed by semi-nested multiplex polymerase chain reaction method (RT-PCR) in two rounds, using type-specific primers for VP7 gene (G1-4, G8-10 types) and for VP4 gene (P[8], P[4], P[6], P[9] and P[14] types) separately, as previously described [26]. PCR products were analysed by agarose gel electrophoresis and ethidium bromide staining.

### Sequence analysis

The partial first-round PCR-derived product of the VP4 and VP7 genes from selected four RV strains circulating in Venezuela during the years 2007/2008 and strains obtained during the year 2003 before vaccine introduction in Valencia (Carabobo state, Venezuela) [26], representative of the main RV genotypes found, were purified using a commercial column (QIAquick PCR purification kit, QIAGEN^®^, Hilden, Germany) and automatically sequenced in both directions using BigDye Terminator cycle chemistry and a 3130XL DNA analyzer (Applied Biosystems, Foster City, CA, USA). A commercially obtained lyophilized dose (Lot n. A41CA419A) of Rotarix^®^ vaccine (GlaxoSmithKline Biologicals, Rixensart, Belgium), used in Venezuela during the 2007-2008 season, was reconstituted according to the manufacturer’s instructions, the viral genome was extracted and amplified as described above, and purified VP4/VP7 first-round PCR amplicons also sequenced.

### Phylogenetic analysis of VP7 and VP4

Alignment and comparison of nucleotide and deduced amino acid sequences of VP7 and VP4 genes from the selected Venezuelan RV isolates were performed by using BLAST^®^ (Basic Local Alignment Search Tool) web service of U.S. National Library of Medicine (blast.ncbi.nlm.nih.gov), and ClustalW method. Phylogenetic and molecular evolutionary analyses were conducted using MEGA version 6.06 [34]. The phylogenetic trees were generated by neighbor-joining method and with the Kimura’s two-parameter as substitution model, from nucleotide sequences of the RV strains in this study together with global reference strains available in the NCBI (National Center for Biotechnology Information) GenBank database (https://www.ncbi.nlm.nih.gov/nucleotide/) and Rotarix^®^ vaccine strain. The statistical significance of the branches was assessed by bootstrap resampling analysis (1000 replicates).

### Protein structure analysis of VP7

A high-quality homology-based model from VP7 deduced amino acid sequences from G1P[8] strains from this study was generated automatically from the ModBase database by the ModWeb server version r181 from the University of California (San Francisco, USA) [35]. The models were built by using the crystal structure (Protein Data Bank identifier [PDB ID], 3FMG) of RV outer capsid protein VP7 trimer in complex with a neutralizing Fab as template [36]. Protein structural analysis and modeling were performed using the UCSF Chimera-Molecular Modeling System v1.11 (University of California, San Francisco) [37], and the Adobe Illustrator CS6 software was utilized as a design tool of the image.

### Nucleotide sequence accession numbers

The VP7 and VP4 gene sequences of the Venezuelan RV isolates reported in this work were deposited in the GenBank nucleotide sequence database under the accession numbers KY039372-KY039373 and KY053848-KY053851 for VP7 gene, and KY056539-KY056544 for VP4 gene.

### Statistical analysis

Data were analysed for the comparisons of prevalence rates using 2x2 tables with χ2 test, or Fisher’s exact test (two-tailed, 95% confidence intervals) when the size sample was less than 5 [Epi Info™ 7.1.4.0, CDC, Atlanta, GA, USA). Student’s test was applied for comparisons of variable values. Tests were considered significant when *p* <0.05.

## Results

### RV detection, PAGE and G/P genotyping

**Fig. 1:**
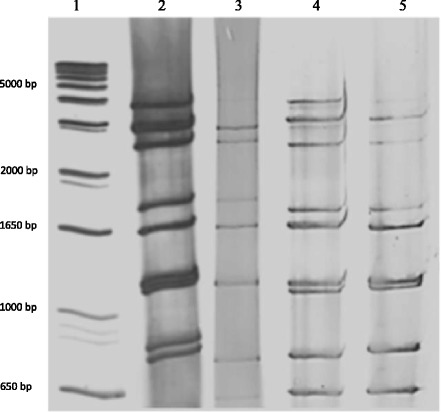
Representative RNA patterns obtained by polyacrylamide gel electrophoresis (PAGE) of rotavirus strains isolated from children with diarrhea from Caracas. Rotavirus RNA segments extracted from fecal samples were separated in a 7% polyacrylamide gel at room temperature for 3 h at 90 V and visualized by staining with silver nitrate as described previously [33]. All the isolates exhibited a profile of group A rotavirus (4-2-3-2). The standard molecular weight (*1 Kb Plus DNA Ladder*, Invitrogen^TM^, CA, USA) (lane 1), one RNA short (slower-moving gene segment 11) (lane 2) and three RNA long (faster-moving gene segment 11) patterns (lane 3–5) of RV genomes are shown

Of a total of 912 stool specimens, 206 (22.6%) rotavirus-positive were detected by immunochromatographic test, of which 169 (82%) were confirmed by PAGE, showing a prevalence rate of RV infection of 18.5%. Based on the electrophoretic migration pattern by PAGE, all the isolates exhibited a profile of RV group A (4-2-3-2) (Fig. 1). Eighty-two (49%) of them showed a long (faster-moving gene segment 11) and 87 (51%) a short (slower-moving gene segment 11) pattern.

**Fig. 2:**
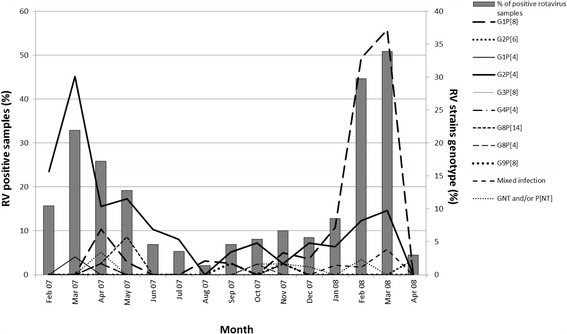
Temporal distribution of rotavirus infections among children with acute diarrhea from Caracas between February 2007 and April 2008. The figure shows the percentage (%) of RV infections (left axis) on the total of diarrhea (*n* = 912) detected among children with acute diarrhea <10 years of age, and the percentage (%) of each RV genotype (right axis) on the total number of RV strains detected per month

A variable detection rate of RV diarrhea was observed during the entire period studied, ranging from 2.1 to 50.1%, with the highest frequency observed during the coolest and driest months, from February to May 2007 and from February to March 2008, and a gradual decline of RV detection from March until August 2007 (Fig. 2, left axis). The comparison between the detection average rate (24%) of 2007 RV peak season (February, March and April) and that of 2008 (36%) revealed a significant increase (*p* = 0.008) of RV diarrhea cases between the two period.

Rotavirus caused diarrhea in children from 7 to 84 months of age, but the most of cases occurred during the first 2 years of life (median age 22 months). The male to female ratio was approximately 1:1. The majority of RV strains (84%) were detected from children who had not received any RV vaccine.

A total of 165 (97.6%) strains were characterized for the G (VP7) and P (VP4) genotype; the remaining four (2.4%) stool specimens were not genotyped because a PCR product could not be generated. The RT-PCR assays revealed that 77 (46.7%) of the 165 strains, were G2P[4], 63 (38.2%) G1P[8], four (2.4%) G8P[14], two (1.2%) G9P[8] and two (1.2%) G1P[4] throughout the study. Additionally, it was found one (0.6%) strain for each one of the following combinations: G3P[8], G2P[6], G4P[4] and G8P[4]. Six (3.6%) samples showed a mixed infection (G1-G2/P[8]-P[4]) and seven (4.2%) were G or P untypeable (NT).

The analysis of genome by PAGE revealed that most (96%) of the G2P[4] strains had a short RNA pattern, among which could be differentiated nine electropherotypes (data not shown) overall very similar, differing only in the migration of dsRNA segments 7 to 9 upon co-electrophoresis. The G1P[8] strains showed predominantly a long RNA pattern, and at least five slightly different electropherotypes were recognized among them (data not shown), evident for the electrophoretic mobility of the dsRNA segments 3, 7 to 9. All the G8, G9, G4 and G3 strains studied showed a long pattern of RNA migration, while the strain G2P[6] had a short profile.

Remarkably, a changing pattern of the G/P type distribution was demonstrated throughout the RV season 2007-2008. Although G2P[4] represented nearly half of the RV strains during the whole study, this genotype was detected almost during the entire period studied and showed a complete predominance over the others during the 2007 RV peak season. The proportion of G2P[4] RV infected children fluctuated around an average of 80% during the first half-year studied, before falling below 10% in the remaining period (Fig. 2, right axis). This significant reduction of their prevalence coincided with an increase in the number of children infected with G1P[8] RVs during the following months. Even though G1P[8] was scarcely represented for most of the year 2007, it emerged to become the dominant genotype since January until March 2008 (Fig. 2, right axis), when prevailed over the others by approximately 67%.

The median age of the children infected with RV G1P[8] and G2P[4] was identical (21 months, *p* = 0.7). The unusual G8P[14] strains detected between April and May 2007, and G8P[4] in October, were collected mainly from children younger than 24 months of age. The other genotypes appeared sporadically (Fig. 2, right axis).

### Nucleotide and deduced amino acid sequence analysis

#### VP7 sequence analysis

**Fig. 3:**
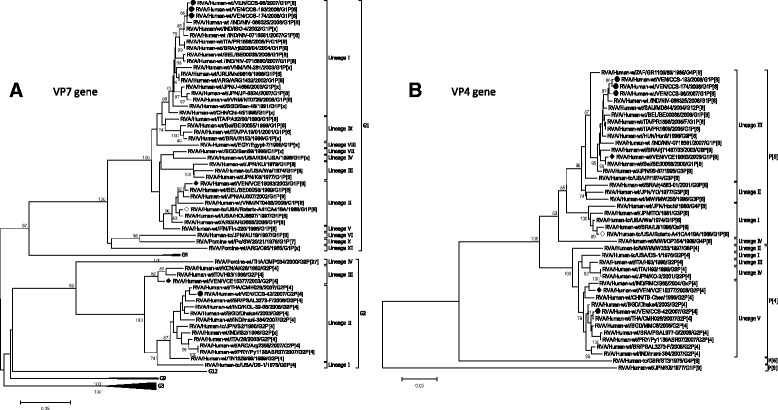
Phylogenetic analysis based on the partial length nucleotide sequence of the rotavirus VP4 and VP7 genes of Venezuelan strains analyzed. **a** Phylogenetic tree constructed from VP7 gene partial sequences (more or less 820 nucleotides). **b** Phylogenetic tree constructed from VP4 gene partial sequences (more or less 890 nucleotides) encoding for the VP8* and part of the VP5* protein subunit. Nucleotide sequences from three G1P[8] and one G2P[4] representative strains of rotavirus circulating in Caracas during 2007-2008 season [marked with a filled circle (●)], were compared with G1P[8] and G2P[4] detected in Valencia (Venezuela) in a previous study [26] accomplished during the 2003 before vaccine introduction [indicated with a filled diamond (♦)], Rotarix®-A41CA419A vaccine strain commercially available [marked with an empty diamond (◊)] and reference rotavirus strains from GenBank database. Rotavirus nomenclature has been used according to the Rotavirus Classification Working Group [68]. For each strain (where available) host species, country of origin, year of isolation and genotypes G-P are shown. The trees were constructed using the neighbor joining method and Kimura’s 2-parameter model. Only bootstrap values above 70%, estimated with 1000 pseudoreplicate data sets, are indicated at each node. Bar is in units of base substitutions per site. The nucleotide sequence data reported in this work were submitted to GenBank with accession numbers KY039372-KY039373 and KY053848-KY053851 for VP7 gene, and KY056539-KY056544 for VP4 gene

Phylogenetic analysis were based on partial sequences of VP7 gene encoding region derived from a PCR amplicon of 884-bp, comprising the hypervariable regions of VP7 protein and surface-exposed amino acids that show intergenotypic variability among prevalent human G and P genotypes (36, 41, 42). Approximately 820 nucleotides of VP7 gene from three randomly selected G1P[8] RV isolates (strains CCS-96/2007, CCS-174/2008 and CCS-193/2008) and one G2P[4] (strain CCS-42/2007) circulating in Caracas during the years 2007-2008 could be analyzed. Additionally two Venezuelan strains, VCE19063/2003 and VCE15377/2003, both detected during 2003 before vaccine introduction, and defined as G1P[8] and G2P[4] respectively in previous genotyping studies [26], were also included for the comparison, as shown in Fig. 3a.  Genotype-specific lineages were assigned as previously described [38, 39].

In the phylogenetic tree of VP7 nucleotide sequences showed in Fig. 3a, the G1P[8] RV strains from Caracas (2007-2008) were grouped in a unique branch, revealing more than 99.8% identities to each other at nucleotide level, and 100% at amino acid level (data not shown). These strains were clustered into the lineage I together with G1 reference strains from India reported in the years 2002, 2007 and 2008 (respectively ISO-4, NIV-0716581 and NIV-088325), for which showed the highest (≥99.5%) nucleotide identities.

**Fig. 4:**
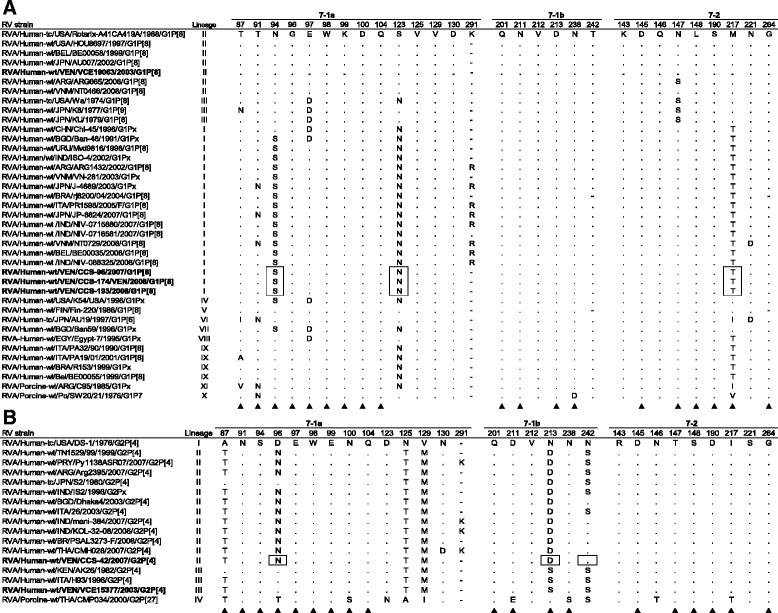
Alignment of amino acid residues defining the neutralization domains in VP7 protein (7-1a, 7-1-b and 7-2) of rotavirus strains analyzed. Deduced amino acid sequences of approximately 270 residues were obtained from rotavirus strains circulating in Caracas (2007/2008) and Valencia (2003) (in bold) and compared with reference strains. For each strain, host species, country of origin, year of isolation and genotypes G-P are shown. Numbering is based on Rotarix^®^-A41CA419A vaccine strain sequence used in Venezuela during the years 2007-2008. The sites not included in the analysis were indicated with a hyphen (-). **a** Neutralization domains from G1 genotype strains analyzed in this study and global reference strains. Identical amino acids with Rotarix^®^ strain in each isolate are identified by dots. Amino acid residue differences among the G1 strains circulating in Venezuela during 2007/2008 season and the pre-vaccine 2003 G1 from Valencia, or Rotarix® vaccine strain, are in boxes. **b** Neutralization domains from G2 genotype strains analyzed in this study and global reference strains. Identical amino acids with the prototype G2 strain DS-1 isolated in 1976 in each isolate are identified by dots. Amino acid residue differences among the G2 strain from Caracas 2007 and the pre-vaccine from Valencia 2003, are in boxes. Amino acid changes that have been shown to escape neutralization with monoclonal antibodies [69] are indicated with a filled triangle (▲)

**Fig. 5:**
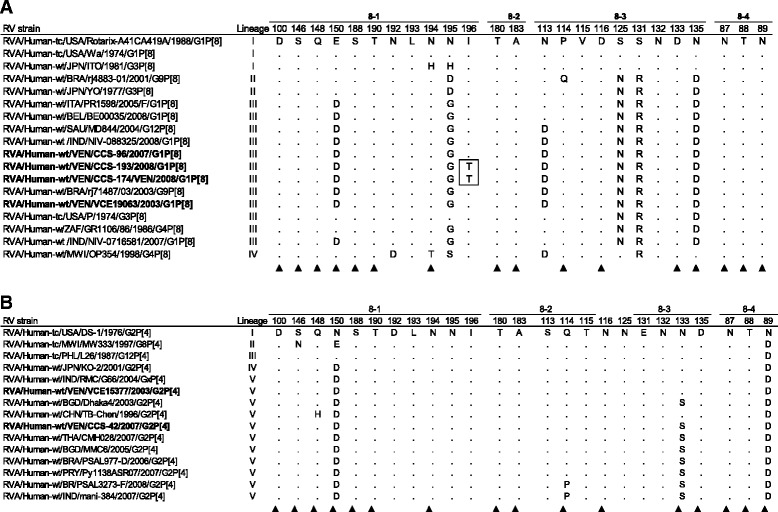
Alignment of amino acid residues defining the neutralization domains in VP8* subunit (8-1, 8-2, 8-3 and 8-4) of VP4 of rotavirus strains analyzed. Deduced amino acid sequences of approximately 330 residues, corresponding to the VP8* and partially VP5* subunit of VP4, were analyzed from rotavirus strains circulating in Caracas (2007/2008) and Valencia (2003) (in bold) and compared with reference strains. For each strain, host species, country of origin, year of isolation and genotypes G-P are shown. Numbering is based on Rotarix^®^-A41CA419A vaccine strain sequence in use in Venezuela during 2007-2008. **a** Neutralization domains from P[8] genotype strains analyzed in this study and global reference strains. Identical amino acids with Rotarix^®^ vaccine strain in each isolate are identified by dots. Amino acid differences exhibited only by the RV P[8] strains from Caracas circulating in 2008, in comparison to any other P[8] strain analyzed, are in box. **b** Neutralization domains from P[4] genotype strains analyzed in this study and global reference strains. Identical amino acids with the prototype G2 strain DS-1 in each isolate are identified by dots. Amino acid changes that have been shown to escape neutralization with monoclonal antibodies [40] are indicated with a filled triangle (▲)

Amino acid differences on the VP7 and VP4 proteins were investigated. A comparison of residues that constituted the epitopes defining the neutralization domains on VP7 and VP4 of the Venezuelan strains G1P[8] and G2P[4], Rotarix® vaccine and other reference strains belonging to the same genotypes, was shown in Figs. 4 and 5. The G1P[8] RV strains from Caracas shared the same amino acid substitutions on the VP7 with Indian strains and other global G1 RVs belonging to the same lineage I and described in geographically distant countries. On the other hand, they showed a lower identity (93%) at both nucleotide and amino acid level to the Rotarix® vaccine strain, which instead was more similar (98%) to the Venezuelan pre-vaccine strain VCE19063/2003 clustered into the lineage II (Fig. 3a). Several amino acid substitutions were observed along the deduced amino acid VP7 sequence of the three 2007/2008 G1P[8] RVs from Caracas in comparison to Rotarix vaccine strain (data not shown), three of them (94N → S, 123S → N and 217M → T) located into the 7-1a and 7-2 neutralization domains within the hypervariable regions of VP7 protein, which were absent in most of the global reference strains belonging to G1 lineages not-I (Fig. 4a). A potential N-linked glycosylation site created by the substitution 123S → N was only observed in the strains of G1-lineage I, including the RV from Caracas, and IX (Fig. 4a). In addition, two substitutions at positions 68A → S and 72Q → R, adjacent to the conserved glycosylation motif Asn-X-Thr (residues 69–71), and others like the mutation 41Y → F, were found on the VP7 of the local strains from Caracas, but not on the Rotarix® and VCE19063/2003 strain (data not shown). No changes were visualized in Ca^++^ binding sites described by Aoki et al. [36] (data not shown).

On the other hand, the strain CCS-42/2007 of G2P[4] genotype detected in Caracas grouped into the lineage G2-II of VP7 gene (Fig. 3a), which showed the highest nucleotide identity to the strains CMH028/2007 (99.2%) reported from Thailand, Dhaka4/2003 (98.6%) from Bangladesh, PSAL3273-F/2008 (98.1%) from Brazil and IND/KOL-32-08 from India (97.9%). A lower identity at nucleotide (92.6%) and at amino acid (95%) level was shown to the Venezuelan pre-vaccine strain VCE15377/2003 (data not shown) that clustered into the lineage III. The deduced amino acid VP7 sequence of the strain CCS-42/2007 from Caracas was very similar to most of the reference strains analyzed belonging to the lineage II, reported before and after vaccine licensure (Fig. 4b). Amino acid differences located into the 7-1a and 7-1b neutralization domains were found at residues 96, 213 and 242 in comparison with the VP7 sequence of the VCE15377/2003 (Fig. 4b).

#### VP4 sequence analysis

Based on the analysis of partial sequences of VP4 gene (more or less 890 nucleotides) encoding for the VP8* and part of the VP5* subunits of VP4 protein, the 2007/2008 G1P[8] RV strains from Caracas exhibited an extremely high genetic similarity among themselves ranging from 99 to 99.8%. They were grouped within the P[8]-III lineage together with the strain VCE19063, detected in Valencia in 2003 before vaccine introduction in Venezuela (Fig. 3b), against which demonstrated a slightly lower nucleotide and amino acid identity, 97 and 98% respectively. Furthermore, VP4 gene of the Venezuelan RV strains showed high percentages of identity (>95.3%) to reference P[8] strains of the same lineage, isolated in different continents during the last two decades (data not shown). A lower identity (around 90%) to the Rotarix^®^ vaccine strain, belonging to the lineage P[8]-I, was found. In fact, the deduced amino acid VP4 sequence analysis revealed several amino acid substitutions along the surface-exposed antigenic epitopes of the VP8* portion defining neutralization domains [40], in comparison to the VP4 of Rotarix^®^ vaccine strain: the substitutions 150E → D and 195N → G within the antigenic region 8-1, and 113N → D, 125S → N, 131S → R and 135N → D into the antigenic region 8-3 (Fig. 5a). Two of the three Venezuelan 2007/2008 G1P[8] strains showed an amino acid change (I → T) at position 196, not related with escape neutralization sites (Fig. 5a) and a unique residue motif K-I-L-V at position 346–349 (data not shown).

The Venezuelan G2P[4] strain CCS-42/2007 isolated from Caracas in 2007 segregated into the P[4]-V lineage (Fig. 3b), sharing a nucleotide identity >98.4% with P[4] global strains of the same period, such as the strains MMC6/2005 and Dhaka4/2003 from Bangladesh, and Py1138ASR07/2007 from Paraguay. Nucleotide and amino acid identity values, respectively of 97.4 and 99%, were found when it was compared to the VCE15377/2003 from Valencia belonging to the same lineage P[4]-V. The Fig. 5b shows the amino acid differences located into the neutralization domains of the VP8* portion of the analyzed strains. The potential trypsin cleavage sites at arginine 240 and 246 were both conserved in all the VP4 sequences studied from Venezuelan RV G1P[8] and G2P[4] strains, as well as the proline at positions 68, 71, 224 and 225, and the cysteine at position 215 (data not shown), residues that have been described as highly conserved in the VP8* gene portion of human RV strains [41].

#### VP7 protein structural analysis and modeling

**Fig. 6:**
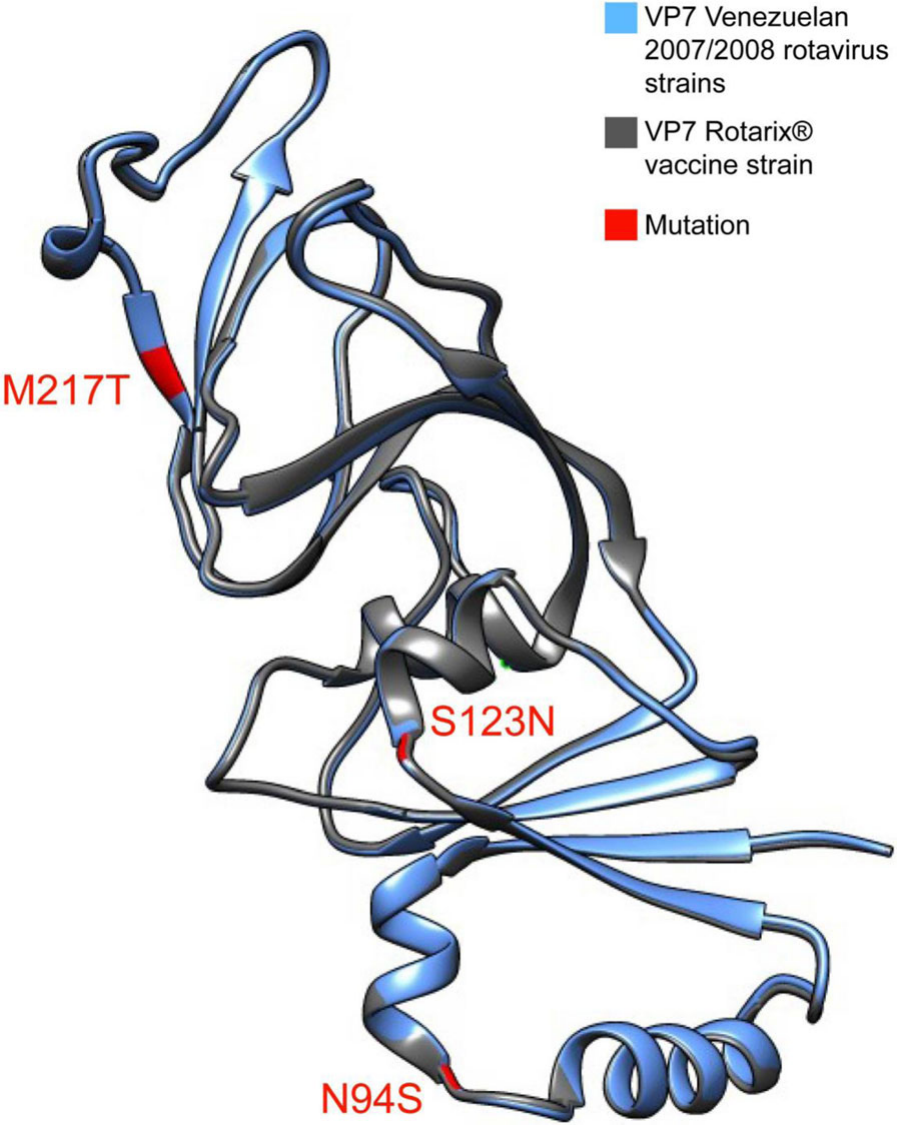
Protein structure analysis and modeling of VP7 from Venezuelan rotavirus G1P[8] strains. The protein structure of VP7 from three Venezuelan G1P[8] strains from Caracas isolated during the season 2007/2008 (*blue*) (represented as just one, because identical among them) has been superimposed to the VP7 protein structure of Rotarix^®^ vaccine strain (*gray*). The model was built using the UCSF Chimera-Molecular Modeling System v1.11 [37]. Surface-exposed residues that differ between the VP7 protein from Venezuelan 2007/2008 RV strains and the Rotarix^®^ vaccine strain are indicated

In order to investigate the effect of the mentioned mutations on the conformation of VP7 protein, an structural analysis comparing the VP7 model from 2007/2008 G1P[8] post-vaccine strains from Caracas and G1P[8] Rotarix® vaccine strain was performed. The analysis showed that both VP7 sequences were perfectly superimposable, where the amino acid substitutions were conservative and did not affect the molecular conformation (Fig. 6). Particularly, no structural modification appeared to have been generated by the three substitutions (94N → S, 123S → N and 217M → T) located into the neutralization domains along the surface-exposed antigenic epitopes on the VP7 of the Venezuelan 2007/2008 G1P[8] strains (Fig. 6).

## Discussion

RV is associated with significant morbidity and mortality rates, particularly in developing countries. Studies performed in Brazil, Mexico, El Salvador and Panama have reported reduction in gastroenteritis and RV disease burden following the introduction of RV vaccines [28, 42, 43], illustrating the health benefits of these strategies. A substantial drop in deaths and hospital admissions after vaccine introduction has been also revealed by some previous works in Bolivia, Honduras and Venezuela [44]. The RV-associated diarrhea rate showed in the present study is remarkably close (>18%) to that reported in Valencia city, not far away from Caracas, some years ago before the introduction of RV vaccines into the national immunization program [45, 46]. This result could be explained by the high percentage of unvaccinated children included in the present study. However, even though the vaccines have been applied in Venezuela since 2006, some authors reported that RV continued causing a considerable number of cases of diarrhea during 2010 [10]. The data showed here might not reflect national and regional trends in rotavirus detection, but the results raise concerns about the factors that could compromise the success of the immunization programs in use in this context.

The efficacy of vaccines in a region can be largely influenced by multiple factors ranging from concurrent enteric infections, malnutrition, immune status, health care access, vaccine coverage rates of the population but also by the RV epidemiology [47]. Although the present study included a relatively short period of time to capture the effects of the seasonality or vaccine introduction, it revealed the occurrence of RV peaks in cooler and drier months. This result contrasts with a previous study performed in the same geographical setting years ago where minimal or absent seasonality was found, but it agrees with the pattern of RV infection observed in Valencia during the years 1998–2002 [45]. The median age of RV infected children included in the present study was higher than that of the children involved in the mentioned study [45], which could explain this difference. It agrees with the association described by other authors between the seasonality differences of RV infection in different regions of a same country and the age of children [48], and with a RV epidemiology systematic review/metaanalysis that reveals a trend of infection to occur in the cool, dry seasons in tropical countries, similar to that observed in temperate zones [49].

On the other hand, concerns exist also about the effects of the vaccination on the RV diversity, considering the selective pressure that RV vaccines could exert on viral populations, leading to changes in the distribution of the genotypes that would compromise the success of the immunization. The assessment of changing pattern of RV infections in children with diarrhea in Caracas during the RV season 2007–2008 after vaccine introduction revealed a distribution of G/P genotypes prevalence similar to that found elsewhere: G1P[8] and G2P[4], two of the five most common RV genotypes found globally during the past three decades [14, 18], were the strains predominantly detected in Caracas. Furthermore, the low proportion of G9P[8], G3P[8], G1P[6] detected ratifies the diversity of the RV genotypes circulating in this geographic region as previously described [23–26]. The occurrence of a few unusual genotype combinations as G8P[14], G1P[4], G4P[4] and G8P[4] was also demonstrated. G8P[14] strains has been found sporadically in humans. In Latin America this genotype has been previously described among RV of animal origin [50] and in humans, specifically in Venezuela from an infant with diarrhea in 2003 [26], and Guatemala from an adult in 2009 [51]. The G8P[14] genotype is believed to have originated from animal RVs that were introduced into human populations through interspecies transmission and/or gene reassortment [50]. Although the G8P[14] strains isolated in Caracas were not able to spread efficiently in the population, as only few strains were detected, further studies are required to understand the transmission potentiality and the origin of these genotype combination that could take part of the complex dynamics of RV evolution.

Extensive global epidemiological surveys have demonstrated that the incidence of rotavirus individual genotypes can show a yearly fluctuation, and multiple rotavirus types can cocirculate within the same region [14, 22, 52]. A temporal variation of the predominant RV genotype occurred in Caracas, where G2P[4] was the RV genotype leading during the 2007 following vaccine introduction in Venezuela, and G1P[8] reemerged during the subsequent months until prevailing in 2008. Predominance of G2P[4] field strains has been repeatedly described almost worldwide concurrently with the introduction of a universal mass vaccination program with the monovalent G1P[8] Rotarix^®^ vaccine [28, 52, 53], but this occurrence has been also seen in the past in countries when had not yet introduced the RV vaccination into the public sector, such as Honduras, Paraguay, Guatemala, Argentina and Bangladesh [14, 52, 54]. Studies performed in Valencia (Venezuela) during the 2003 showed a pattern of fluctuation of RV genotypes similar to that described in the present study, where the higher prevalence of G2P[4] strains was unrelated to RV vaccination [26]. Some authors have found findings suggesting that the relative high frequency of G2P[4] reported in several Latin American countries can reflect a regional phenomenon or a cyclic pattern of RV strains instead of the selective pressure created by the implementation of RV monovalent vaccine [54, 55].

Comparison of the VP7 gene sequence of the Venezuelan G2P[4] strains revealed that RV from Caracas was more similar to global strains of the lineage G2-II, described in old and recent times, than to the pre-vaccine strain from Valencia. The data do not allow determining the precise evolutionary relationship among RV isolated before and after vaccine. Nevertheless, some variations observed after analysis of the deduced amino acid sequence of VP7 protein, and to a lesser extent of VP4, of RV G2P[4] detected after vaccine introduction in Venezuela, arouse interest, particularly two substitutions located into the 7-1a and 7-1b neutralization domains: the 96D → N substitution, which implied a radical change from a negatively charged (aspartic acid) to an uncharged (asparagine) amino acid, and the 213N → D, capable to confer a negatively charged residue (aspartic acid). Moreover, other two amino acid changes observed on the VP4 of this isolate, the 150N → D and the 89N → D, substituted both a polar/uncharged with a polar/negatively charged amino acidic residue. It remains uncertain if such electrical changes on the outer capsid viral protein could have conditioned somehow the spread of the G2P[4] strains into the population in Caracas. The substitution 96D → N on the VP7 has been strongly related in the last decade by other authors to an abrupt increase or reemergence of G2 strains in different European, African and Asian countries, as United Kingdom, Nepal, South Africa, Taiwan, Thailand, Bangladesh and Japan [39, 56–60].

The predominance of G2P[4] genotype in Caracas during the 2007 and its consecutive decline (from 30.1% to <10%) to be overcome by G1P[8] during 2008, is a trend that was also described in other countries as Nicaragua some years ago before vaccine introduction [61]. This event could have been result of differential virus fitness among susceptible and immunological protected hosts. Interestingly, no significant difference in the median age of the G1P[8] and G2P[4] infected children was observed in Caracas, suggesting that other factors beyond the age would be conditioning the host susceptibility to the infection. Following the introduction of the vaccine in a region, different selective pressures can be exerted on the viral populations by the homotypic and heterotypic immunity vaccine-induced. It has been extensively shown that the monovalent G1P[8] Rotarix^®^ vaccine used worldwide confers homotypic and heterotypic protection, providing good and sustained protection against all encountered non-G1 strains (G3, G4, G9) sharing the same P type (P[8]) [2, 4, 62, 63]. Although it may be less efficacious against the G2 strains, an immune response can be achieved through cross-reactive epitopes on VP7 and VP4 proteins, together with VP6 or non-structural proteins epitopes [64]. Thus, the results suggest that the combination of the weaker natural or vaccine-induced immunity against G2P[4] and the natural fluctuations of this genotype could have favored the subsequent reemergence of G1P[8] strains observed in Caracas at the end of 2007, as proposed by other authors [65].

The complete predominance of G1P[8] strains here described over the other types during the following months (early 2008) was unexpected. Since a large proportion of children included in the present study was unvaccinated, it could be assumed they were highly susceptible to be infected by G1P[8] strains circulating at that time. On the other hand, these strains could have acquired some selective advantage favoring their transmission. In fact, the phylogenetic analysis of the VP7 revealed that the G1P[8] strains circulating in Caracas following RV vaccine introduction were grouped into a single genetically homogeneous clade of lineage G1-I, very close to contemporary strains described in geographically remote countries, but they were rather distantly related to Rotarix^®^ vaccine and the Venezuelan pre-vaccine 2003 G1P[8] strain, belonging both to the lineage G1-II. Thus, the post-vaccine G1P[8] strains could have accumulated mutations and evolved over time into a variant that might escape from vaccine induced antibodies. It is difficult to establish when this variant arose, considering that data on the RV strains circulating in nearest pre-vaccination period are not available, but it cannot be excluded that it might have been introduced in Venezuela from other countries or were vaccine-derived.

Studies with animal and human RVs have demonstrated that neutralizing antibodies against VP7 protein play a critical role in vaccine-mediated immunological protection against RV disease, as well as the VP8* subunit of VP4 protein participates in viral infectivity and neutralization [66]. Amino acid substitutions in outer capsid proteins could thus result in escape of viruses from neutralizing antibodies, affect viral fitness, and/or change receptor preference. The Venezuelan G1P[8] post-vaccine strains showed amino acid differences on the 7-1a and 7-2 antigenic epitopes of the VP7 protein that have been also described by other authors on current global G1 strains [67]. Here, the modifications observed were: i) the mutation 217M → T into the domain 7-2 that changed a methionine (non-polar/hydrophobic) with a tyrosine (polar/uncharged), probably defining the lineage G1-I; ii) the 68A → S, which determined a change from an alanine (non-polar, small) to a serine (polar/neutral), adjacent to a conserved glycosylation motif located on the residues 69 to 71; and, iii) a potential N-linked glycosylation site created by the mutation 123S → N, which was absent on the VP7 of Rotarix® vaccine strain and other G1 strains not belonging to the lineage I or IX, whose far ranging effect on the antigenicity of this epitope is unknown. Previous analysis has revealed that the amino acid residues located in the 7-1a and 7-2 antigenic epitopes are distributed fairly heterogeneously across the face of the VP7 molecule, defining the neutralization domains [67]. Modeling structural analysis of the VP7 from Venezuelan G1P[8] strains did not showed differences in the molecular conformation of these epitopes when compared with the VP7 from the Rotarix^®^ vaccine strain, suggesting that the mentioned amino acid mutations did not alter the conformation of the neutralization domains. Nevertheless, although they were structurally conservative mutations, other types of studies are needed to understand whether the changes in electric charges described into the immunodominant regions could have affected the protein-antibody binding and led to loss of vaccine-induced protection.

VP4 analysis revealed that the RVs G1P[8] analyzed, circulating in Caracas after vaccine implementation, were very similar to the G1P[8] pre-vaccine strain VCE19063/2003 from Valencia, which was grouped in the same genetic lineage P[8]-III, but differed from Rotarix^®^ vaccine strain in some amino acid substitutions located into neutralization domains 8-1 and 8-3. Among them, the 196I → T was exclusively present on the VP4 of the G1P[8] strains isolated from Caracas in 2008 (Fig. 5a) and replaced a non-polar/hydrophobic with a polar/uncharged amino acid, determining a change of electrical charge.

## Conclusions

Antigenic variants of viruses are expected to emerge as the population immunity evolves. The emergence and later predominance of G1P[8] strains observed in Caracas during the 2007-2008 RV season and described in this study suggests that these RV could have escaped from the immune response evocated against strains previously circulating in the region. The observed amino acid changes on the outer capsid proteins VP7 and/or VP4, which determined alterations in the electrical charges of residues located onto the neutralization domains, could have subtly affected the binding of neutralizing antibodies and conferred a selective pressure influencing the viral fitness, and favoring the transmission of the viruses. Thus, the result of forces and balances that drive rotavirus natural evolution would determine the spread of novel strains. The impact that the widespread use of RV vaccines will have on the diversity and evolution of human RVs is hardly predictable. The vaccination continues being the best control strategy against the RV diarrhea, thus it is important to achieve higher vaccine coverage rates in countries as Venezuela. Although official data are not available, it is presumed that the current coverage for oral RV vaccines in this country would surpass 75%. Therefore it is expected that vaccine impact may intensify in the next years.

This study highlights the importance of monitoring the transitions in the prevalence of genotypes and understanding of their effect on the efficacy of currently available vaccines. The genotype prevalence changes described after vaccine introduction in Caracas raise concerns, but such variations should be interpreted cautiously in the global context over time of a determined geographic area. Additionally, a continued surveillance of the genetic characteristics of RVs circulating will continue to be needed to obtain a better view of the long-term effects of vaccine introductions, to assess intra-genotype evolution that may lead to selection for strains that could escape homotypic immunity from the vaccine or that are antigenically different from those included in the vaccine formulations, and to determine the potential of their global spread. Since the immunogenicity and efficacy of the RV vaccines may be challenged by evolution of the viral genome of RV circulating, it is also important to accomplish the full genome analysis of strains collected in different time or geographic regions of a same country and know their evolutionary profile during post-licensure surveillance.

## Abbreviations

BLAST: Basic Local Alignment Search Tool
dsRNA: Double strand RNA
G: VP7
MEGA: Molecular Evolutionary Genetics Analysis
NCBI: National Center for Biotechnology Information
NT: Untypeable
P: VP4
PAGE: Polyacrylamide gel electrophoresis
PDB: Protein Data Bank
RT-PCR: Reverse transcriptase polymerase chain reaction
RV: Rotavirus
